# Cure Cycle Optimization of Rapidly Cured Out-Of-Autoclave Composites

**DOI:** 10.3390/ma11030421

**Published:** 2018-03-13

**Authors:** Anqi Dong, Yan Zhao, Xinqing Zhao, Qiyong Yu

**Affiliations:** School of Materials Science and Engineering, Beihang University, Beijing 100191, China; dong20121226@gmail.com (A.D.); zhaoxinqing201314@163.com (X.Z.); yqy_318@163.com (Q.Y.)

**Keywords:** out-of-autoclave, rapidly cure, porosity, microstructure, fracture surface

## Abstract

Out-of-autoclave prepreg typically needs a long cure cycle to guarantee good properties as the result of low processing pressure applied. It is essential to reduce the manufacturing time, achieve real cost reduction, and take full advantage of out-of-autoclave process. The focus of this paper is to reduce the cure cycle time and production cost while maintaining high laminate quality. A rapidly cured out-of-autoclave resin and relative prepreg were independently developed. To determine a suitable rapid cure procedure for the developed prepreg, the effect of heating rate, initial cure temperature, dwelling time, and post-cure time on the final laminate quality were evaluated and the factors were then optimized. As a result, a rapid cure procedure was determined. The results showed that the resin infiltration could be completed at the end of the initial cure stage and no obvious void could be seen in the laminate at this time. The laminate could achieve good internal quality using the optimized cure procedure. The mechanical test results showed that the laminates had a fiber volume fraction of 59–60% with a final glass transition temperature of 205 °C and excellent mechanical strength especially the flexural properties.

## 1. Introduction

Out-of-autoclave (OOA) composite has rapidly developed in the aerospace industry due to no limitation on part dimensions and lower cost as well as the comparable mechanical properties with autoclave composite. However, maximum consolidation pressure is only one bar in the out-of-autoclave process. As a result, the air evacuation and quality guarantee become the critical factors to produce void-free composite parts. Typically, out-of-autoclave prepregs are manufactured by applying resin films on the two outer surfaces and partially impregnated leading to dry fiber areas in the center [1]. The particular microstructure is beneficial to remove entrapped air within the prepreg stacking at room temperature. However, the porous areas need to be completely infiltrated by resin during the cure step to obtain porosity free parts. It requires the out-of-autoclave resin to have a different behavior with the autoclave resin. At room temperature, the reactivity of the out-of-autoclave resin should be lower to avoid cold flow and maintain the porous areas for air evacuation while at elevated temperature, the viscosity of resin needs to decrease rapidly to impregnate the porous areas prior to gelation. It is important to balance the resin flow time and gel time. Otherwise, voids may exist inside [2]. Therefore, dwelling at a proper temperature is a necessary step to give sufficient time for resin to impregnate the porous areas before gelation [3,4]. In the earlier study, the curing reaction of out-of-autoclave resin occurred too quickly, which is why the resin flow time reduced and resulted in voids [5]. Therefore, thermal behaviors of out-of-autoclave resin need to be deeply analyzed to predict the heat generation in the composite parts especially in the thick ones. Inappropriate heat generation by resin curing reaction may cause a temperature gradient for the temperature inside may be higher [6,7]. Meanwhile, it is important to understand the viscosity evolution to monitor the process of resin infiltration and obtaining porosity free parts [8,9].

Meanwhile, a free-standing post-cure step is required to further improve the degree of cure, which is similar to traditional thermoset resins [2,10,11]. The duration of post-cure correlates to the final glass transition temperature as well as the mechanical properties of out-of-autoclave composite [12,13]. In addition, it should be noted that in the curing step, the glass transition temperature should be above the cure temperature especially for large and contoured structure. Otherwise, the part may deform after it is removed from the tool [14,15].

Based on the above reasons, the manufacturing time for out-of-autoclave composite usually varies between seven and seventeen hours such as traditional out-of-autoclave materials CYCOM 5320-1 [11,16]. It is essential to reduce the manufacturing time in order to achieve real cost reduction and take full advantage of the out-of-autoclave process [17,18].

This study aims to reduce the cure cycle time and production cost while maintaining high laminate quality. A rapidly cured out-of-autoclave resin was independently developed by our team. Based on the resin, the typical partially impregnated out-of-autoclave prepregs reinforced with T800 carbon fibers were also manufactured by a traditional two-step process. To determine a suitable rapid cure procedure for the developed prepreg, the effect of heating rate, initial cure temperature, dwelling time, and post-cure time on the final laminate quality was evaluated and the factors were optimized. As a result, a rapid cure procedure was determined. To validate the cure procedure, resin impregnation evolution during the cure procedure was studied and the microstructure of the prepregs stack during cure cycle was observed by scanning electron microscope. The degree of cure, viscosity, and glass transition temperature evolution of the developed out-of-autoclave prepreg during the cure procedure were also summarized and compared with those of the traditional out-of-autoclave prepreg. Moreover, mechanical properties of the developed out-of-autoclave composites cured according to the determined cure cycle were examined. For comparison, mechanical performance of traditional out-of-autoclave composite and autoclave composite were also tested.

## 2. Materials and Methods

### 2.1. Materials

**Figure 1:**
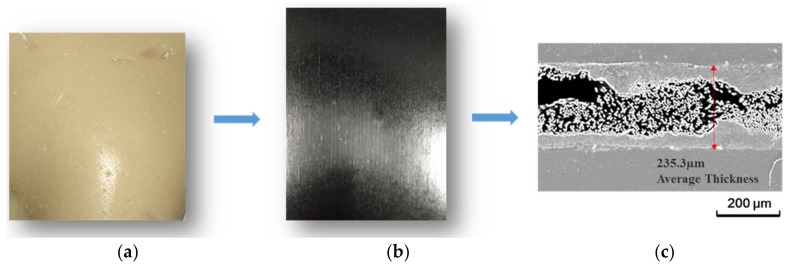
(**a**) The developed out-of-autoclave resin; (**b**) unidirectional prepreg manufactured with developed out-of-autoclave resin; (**c**) optical micrographs for the prepreg.

An independently developed out-of-autoclave resin was used in this study, which could be cured rapidly compared to the traditional out-of-autoclave resin. This resin system contained multifunctional phenolic epoxy resins, which were toughened by polyethersulfone. Diaminodiphenysulfone was used as the curing agent in this resin system. The epoxy equivalent of the resin was 0.5. The percentage of the thermoplastic modifier in the resin was 10%. The mass ratio of epoxy and DDS was 100:31. Prepregs based on the developed resin reinforced with T800 carbon fibers were prepared by a traditional two-step process. Fiber volume fraction of the prepregs was 60%. Fiber area weight was 145 g/m^2^. The prepregs were partially impregnated with dry fiber areas in the center as the passage for air evacuation. Figure 1a,b show the optical picture of the developed resin and prepreg, respectively. Figure 1c shows the microstructure of the prepreg. For comparison, CYCOM5320-1 resin and relevant prepregs were also used, which were traditional out-of-autoclave materials.

### 2.2. Degree of Cure

The degrees of cure measurements were conducted on a Mettler DSC822e instrument (Zurich, Switzerland). In isothermal DSC measurements, 100 °C, 120 °C, 140 °C, 160 °C, 180 °C, and 200 °C were selected as the isothermal temperature. The ‘heat-cool-heat-cool’ program was set. First, samples were quickly heated to desired isothermal temperature and stayed long enough to achieve constant heat flow. Then the samples were cooled to room temperature, which was afterward followed by heating again to 350 °C at a heating rate of 2 °C/min to measure the residual heat. Finally, the reaction heat for each isothermal temperature was calculated based on each residual heat and total reaction heat. The degree of cure was calculated according to Equation (1) [19,20].

*α* = (*H_total_* − *H_r_*)/*H_total_*, (1)

where *α* is degree of cure, *H_total_* is the total reaction heat, and *H_r_* is the residual reaction heat. The degree of cure along with the cure cycle was also tested at fast (5 °C/min) and slow (1.5 °C/min) heating rates, respectively. The weight of samples was 7–10 mg. All DSC measurements were conducted in nitrogen atmosphere with a flow rate of 50 mL/min.

### 2.3. Viscosity

Viscosity measurements were conducted on a TA advanced rheometer 2000 (TA Instruments, New Castle, DE, USA) using 25 mm-diameter parallel plates with the frequency of 10 rad/s. In each test, the resin sample around 1.5 g was used to investigate the influence of heating rates on the viscosity of the resin.

### 2.4. Glass Transition Temperature

Glass transition temperature measurements were conducted on a TA Q800 DMA instrument (TA Instruments, New Castle, USA). The testing mode was three-point bending. The dimension of samples was 40 mm × 8 mm × 2 mm. The samples were tested with 80 µm sinusoidal displacements at a frequency of 1 Hz. The test temperature range was 25–300 °C [21]. The temperature rate was 5 °C/min.

### 2.5. Thermogravimetric Analysis

**Figure 2:**
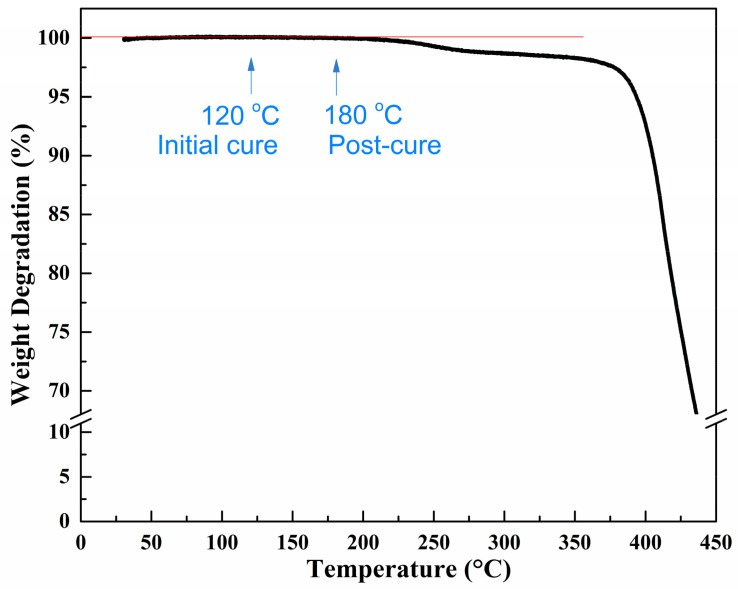
Thermogravimetric curve of the prepreg.

The thermogravimetric analysis was carried out under dynamic condition on a TGA-50/50H instrument (Shimadzu, Kyoto, Japan) from 25 °C to 450 °C at a heating rate of 5 °C/min. The weight of sample was 7 mg. The nitrogen was involved as purge gas to simulate the real vacuum condition in out-of-autoclave process with a flow rate of 50 mL/min. The test result is shown in Figure 2, which indicates no weight degradation under 200 °C for the developed prepreg. Therefore, the effect of volatiles and moisture on void formation of composites could be neglected. In this study, the formation of voids in the final laminate part was considered to be insufficient air evacuation.

### 2.6. Void Content and Microstructure

The void content and microstructure were studied by a Leica DM4000 optical microscope (Wetzlar, Germany). To calculate the void content, the samples from different locations of the laminates were mounted in epoxy resin and then grounded and polished to get a scratch-free surface. The micrographs of the sample were obtained under the optical microscope. The void content of the sample was calculated as an average value from at least 20 micrographs.

### 2.7. Mechanical Properties Evaluation

The mechanical properties were tested on an Instron 5967 instrument (Norwood, MA, USA). The testing standards used for tensile, compression, flexural, and short-beam shear tests were ASTM D3039, ASTM D6641, ASTM D7264, and ASTM D2344 [22]. The fracture mode and fracture surface of samples were examined by using a JSM6010 scanning electron microscope (Tokyo, Japan).

## 3. Results and Discussion

### 3.1. Effect of Heating Rate

#### 3.1.1. Degree of Cure

**Figure 3:**
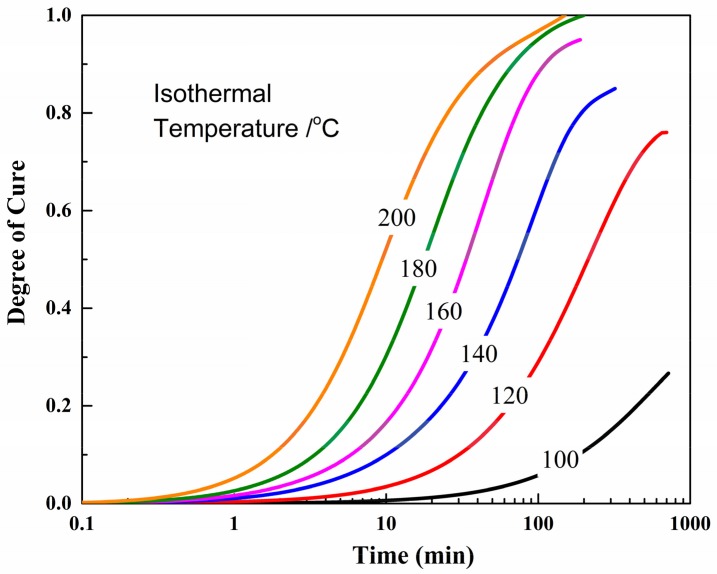
Degree of cure evolution at various isothermal temperatures.

The degree of cure represents the cross-linking degree of resin. It is essential to ensure complete cure of the final composite laminates, which guarantees a good laminate quality [23]. Figure 3 lists the degree of cure evolution at various isothermal temperatures for the developed out-of-autoclave resin. It was shown that the degree of cure increased with cure time until reaching a constant value, which was the maximum degree of cure obtained at that temperature. As the isothermal temperature grew, the maximum degree of cure increased. The maximum degree of cure at 120 °C and 140 °C was 0.78 and 0.85, respectively, which suggests an insufficient cross-linking network formation. As mentioned above, the out-of-autoclave resin needed to be further cured at the post-cure stage to achieve good quality. As shown in Figure 3, when the temperature was raised to 180 °C, the cure degree of resin reached 0.95. The results indicated that 180 °C could be selected as the post-cure temperature.

**Figure 4:**
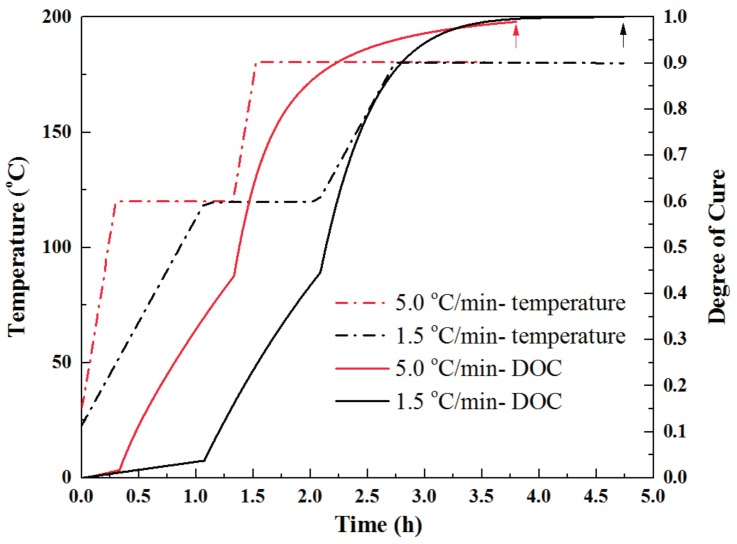
Degree of cure as a function of cure cycle time for fast and slow heating rate.

We selected 1.5 °C/min and 5 °C/min to study the effects of heating rates on the degree of cure. The heating rate of 1.5 °C/min was commonly recommended by the out-of-autoclave resin manufacturer. The cure cycle was composed of two stages including an initial cure stage of dwelling at 120 °C for 1 h and a post-cure stage dwelling at 180 °C for 2 h. As shown in Figure 4, after ramping to the initial cure temperature, the slow heating rate showed a slightly higher degree of cure, but still lower than 0.10. It was logical that the sample was held for a longer time (63 min) at the slow heating rate during the first temperature-rise period, which improved the resin cross-linking degree. During the initial cure stage, a similar trend of cure degree evolution was observed for both fast and slow heating rates. Figure 4 also showed after dwelling at post-cure stage for 2 h, the resin could be completely cured for both fast and slow heating rates. The results suggested that the fast heating rate was more efficient and saved 35% cure time compared to the slow heating rate. The resin could be completely cured as well.

#### 3.1.2. Viscosity

**Figure 5:**
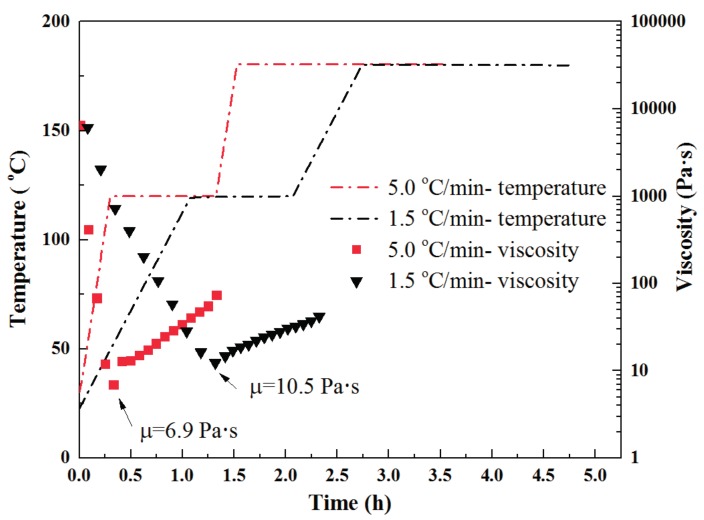
Viscosity evolution during the cure cycle for fast and slow heating rate.

As mentioned in the materials section, the out-of-autoclave prepregs were partially impregnated to help with air evacuation at room temperature. However, the dry fiber areas needed to be infiltrated by the low viscosity resin at the initial cure stage [24,25]. To evaluate the effect of heating rate on the viscosity of resin and avoid insufficient resin impregnation, the viscosity of the developed resin during the cure cycle time at the heating rates of 1.5 °C/min and 5 °C/min were tested, which is shown in Figure 5. Regardless of the heating rate, the viscosity exhibited a similar trend during the heating and initial cure stage. The minimum viscosity for the fast and slow heating rates were 6.9 and 10.5 Pa·s, respectively. Both were suitable for resin infiltration. After dwelling at initial cure stage for 1 h, the resin still exhibited relative lower viscosity and there was no sign for gelation, which contributed to resin infiltration. In addition, many researchers have suggested that the fast heating rate could induce lower resin viscosity and could be beneficial to resin infiltration [26,27,28]. However, in this study, no disadvantage of the slow heating rate was observed for the resin viscosity.

### 3.2. Effect of Initial Cure Temperature

**Figure 6:**
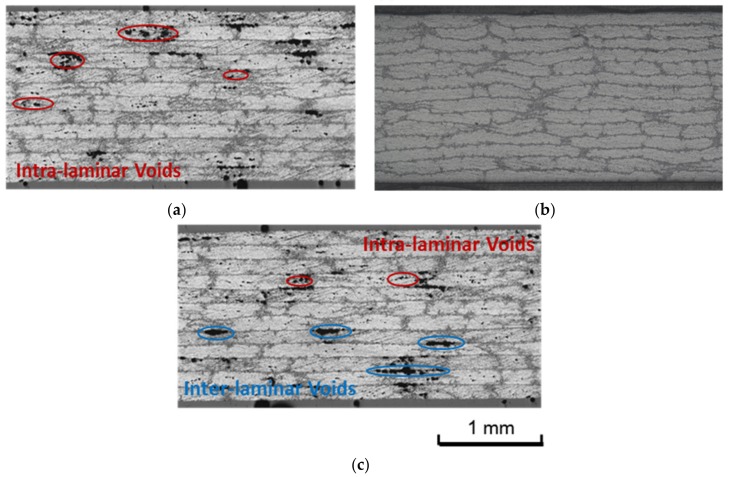
Optical micrographs for laminates cured with various initial cure temperature: (**a**) dwelling at 100 °C for 1 h; (**b**) dwelling at 120 °C for 1 h; and (**c**) dwelling at 140 °C for 1 h.

**Figure 7:**
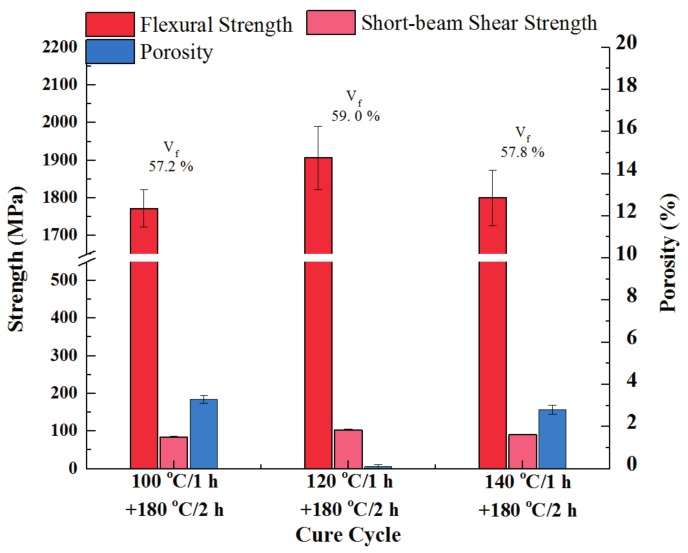
Effect of initial cure temperature on mechanical properties and void content of laminates.

In order to evaluate the effect of the initial cure temperature and determine the suitable cure cycle, various initial cure temperatures were employed to cure the developed out-of-autoclave prepreg stack. Stacking sequence of [0]_16_ was selected for all laminates. Regardless of the initial cure temperature, all of the laminates were 1 h dwell at the initial cure stage, which was followed by dwelling at 180 °C for 2 h while the heating rate was 5 °C/min during the whole process. The laminates were cured in the oven, which is associated with vacuum pressure. After curing, the microstructures, flexural strength, short-beam shear strength, and porosity of the final laminates were evaluated, which is presented in Figure 6 and Figure 7. A notable difference in porosity was observed. When the initial cure temperature was 100 °C, obvious intra-laminar voids could be seen in the final composite with a statistic porosity of 3.3%. Considering partially impregnated structure of the initial out-of-autoclave prepreg, which was beneficial to the air evacuation under only one atmospheric pressure, the resin viscosity was identical for resin impregnation. The minimum resin viscosity of 30 Pa·s at 100 °C for the developed resin was not low enough for infiltrating each prepreg layer. It hindered the air evacuation and led to the formation of voids. The intra-laminar voids caused the decrease in flexural strength and short-beam shear strength. On the contrary, the viscosity at 140 °C was low, but the gelation reaction occurred too fast, which led to insufficient resin impregnation. Both inter-laminar and intra-laminar voids could be seen in the micrograph and the total void content was 2.8%. When the composite was initially cured at 120 °C, both flexural strength and short-beam shear strength of the final part reached a maximum value, 1906.2 MPa and 103.5 MPa, respectively, and there was no significant void in the laminates. In addition, the laminates initially cured at 120 °C had a maximum flexural modulus of 150.23 GPa. While the flexural modulus of laminates cured at 100 °C and 140 °C were 143.88 GPa and 148.61 GPa, respectively. Hence, 120 °C is suitable as the initial curing temperature for the developed resin.

### 3.3. Dwell Time at Initial Cure Stage

**Figure 8:**
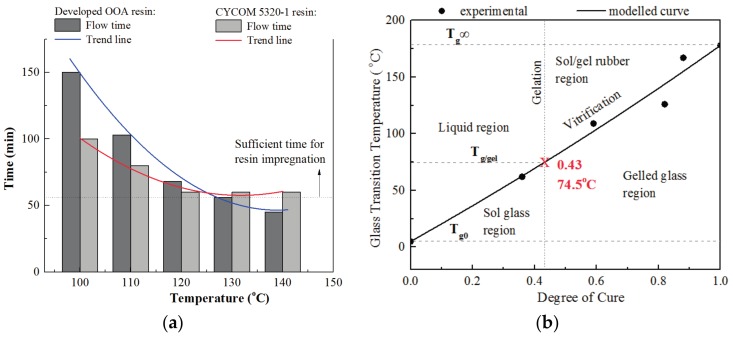
(**a**) Flow time of rapidly cured laminates at various isothermal temperatures; (**b**) Gelation conversion of the developed resin.

As the aim of the initial cure stage was to infiltrate dry fiber areas by resin completely, the sufficient flow time for the resin should be set to guarantee good quality. Figure 8a listed the flow time at various isothermal temperatures for the developed resin and was compared to the traditional out-of-autoclave resin CYCOM 5320-1 as well. Figure 8b showed the gelation conversion of the developed resin. The resin flow time of CYCOM5320-1 resin was more than 60 min and it could prevent void defects in the final composite parts [8]. For the developed resin, the flow time was sufficient for infiltration below 120 °C. However, when the temperature grew to 140 °C, the gelation occurred after only 45 min as a result of higher reactivity of resin, which implies that dwelling at 140 °C was not enough for the resin impregnation. The results also confirmed that 120 °C was suitable as the initial curing temperature.

### 3.4. Effect of Post-Cure Time

**Figure 9:**
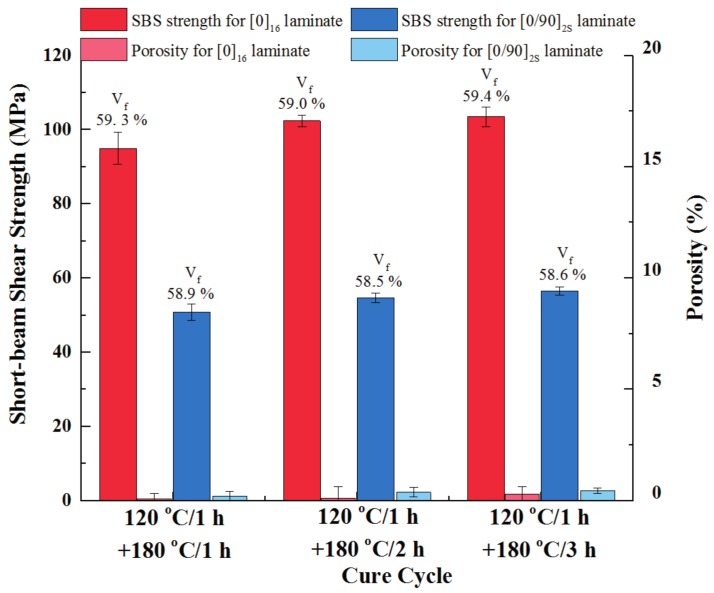
Effect of post-cure time on mechanical properties and void content of laminates.

For the post-cure stage, the developed out-of-autoclave resin dwelling at 180 °C can be cured completely, which was proven above. However, on the premise of good laminate quality, it is beneficial to reduce the dwelling time in order to save the manufacturing cost. Therefore, various post-cure durations were employed to cure the developed out-of-autoclave prepreg stacks. Stacking sequences of [0]_16_ and [0/90]_4S_ were both selected. Regardless of the post-cure time, all of the laminates were cured at 120 °C for 1 h, followed by a post-cure stage at 180 °C during which the heating rate was 5 °C/min across the whole process. The laminates were cured in the oven and were associated with vacuum pressure. After curing, short-beam shear strength and porosity of the final laminates were evaluated, which is presented in Figure 9. The results indicated that, after dwelling at 120 °C for 1 h, all laminates exhibited the minimum porosity less than 1.0%, which is consistent with the above conclusion. As for the short-beam shear strength, the strength of unidirectional laminates after post-curing for 2 h was 102.4 MPa, which is an increase of 7.8% compared to that of laminates after post-curing for 1 h (95 MPa). For the cross-ply laminates, the strength of laminates after post-curing for 2 h was 54.7 MPa, which increased by 7.7% compared to that of laminates post-curing for 1 h (50.8 MPa). However, when the duration of post-curing time further increased to 3 h, the short-beam shear strength of both unidirectional and cross-ply laminates was 103.5 MPa and 56.5 MPa, respectively, which shows no significant improvement when compared to that of laminates after post-curing for 2 h. Meanwhile, the fiber volume fraction of the laminates was close after post-curing for different time periods. It was concluded that the optimum dwelling time at 180 °C was 2 h, which reached the balance between laminate quality improvement and manufacturing cost saving.

**Figure 10:**
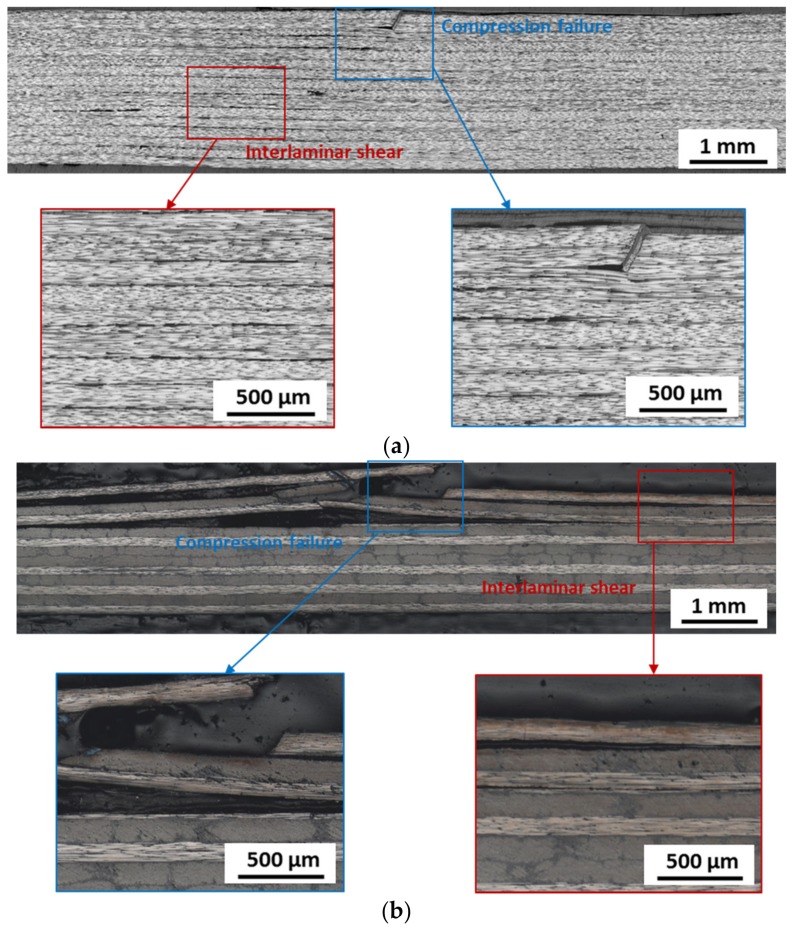

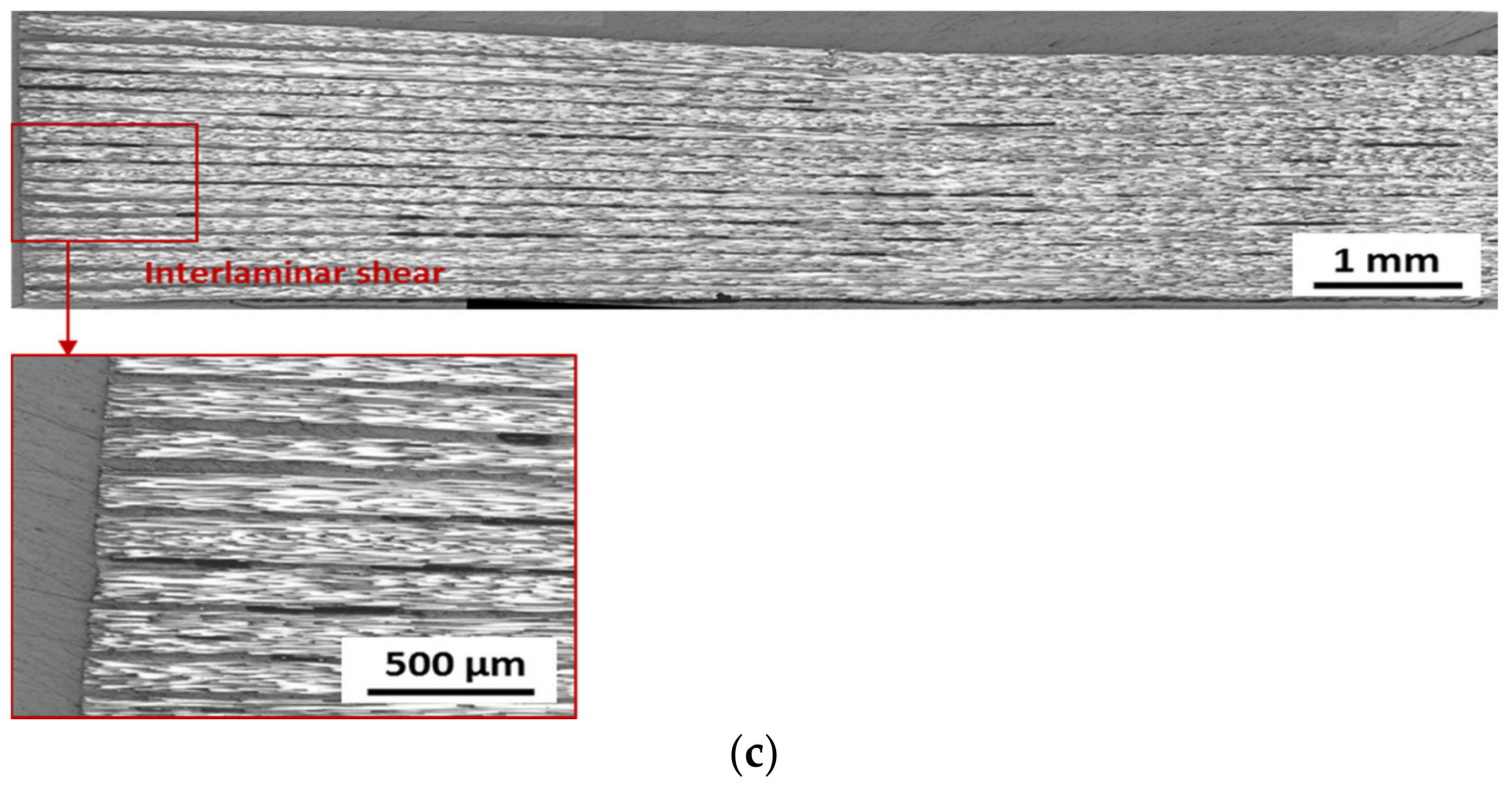
Fracture modes in short-beam shear tests of laminates post-cured for various time: (**a**) unidirectional laminate after post-curing at 180 °C for 2 h; (**b**) cross-ply laminate after post-curing at 180 °C for 2 h; and (**c**) unidirectional laminate after post-curing at 180 °C for 1 h.

Typically, there are four kinds of failure modes in the short-beam shear test according to ASTM D2344 standard including interlaminar shear, compression failure on the top surface, tension failure on the bottom surface, and inelastic deformation [29]. The fracture modes of the developed out-of-autoclave laminates post-cured for different times was observed, which is shown in Figure 10. Both compression failure and interlaminar failure were observed in the laminates of unidirectional and cross-ply stacking sequence after post-curing for 2 h. In contrast, only the inter-laminar shear failure mode was found in the laminate post-curing for 1 h. Compression failure indicated high fiber-matrix interfacial strength and interlaminar strength induced by fiber breakage on the top surface. This suggested that the short-beam strength was not only dominated by resin, but also the bonding between fiber and matrix. However, for the laminates after post-curing for 1 h, the short-beam shear strength was dominated by the weak interlaminar strength, which led to the initial interlaminar shear failure.

**Figure 11:**
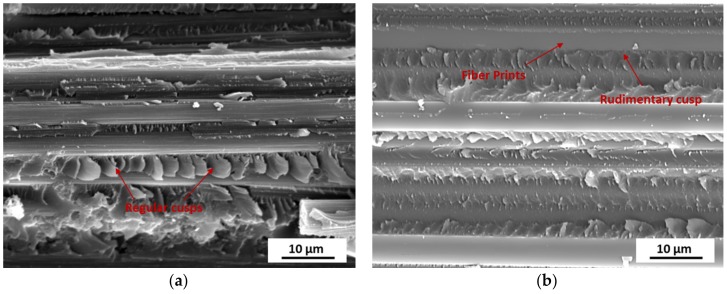
SEM images of fracture surface in short-beam shear tests of laminates post-cured for various time: (**a**) unidirectional laminate after post-curing at 180 °C for 2 h; (**b**) unidirectional laminate after post-curing at 180 °C for 1 h.

Figure 11 shows the SEM images of the fracture surface of laminates in short-beam tests. For the laminate post-cured for 2 h, significant regular cusps were observed on the fracture surface as well as the parallel fracture defects, which suggests strong fiber-matrix interfacial bonding. In addition, there remained resin on the fiber surface, which also proves good interface adhesion. No obvious fiber misalignment existed in the fracture surface. As for the laminate post-cured for 1 h, it exhibited a much smoother fiber surface as the result of weak fiber-matrix bonding. Generally, it was reported that the micro-crack initially appeared in the resin in short-beam shear tests at a 45° angle to the fiber direction and gradually expanded to the fiber-matrix interface. Then it aggregated together. Once the fiber-matrix interfacial strength was improved, the obvious parallel fracture defects could be detected [30].

### 3.5. Examination of Laminate Quality

From the above results, a rapid cure cycle was determined for the developed out-of-autoclave composites, which was dwelling at 120 °C for 1 h and followed by dwelling at 180 °C for 2 h at a heating rate of 5 °C/min during the whole process. To further study the resin impregnation evolution and ensure good laminate quality, the microstructure of the prepregs stack during cure cycle was monitored.

**Figure 12:**
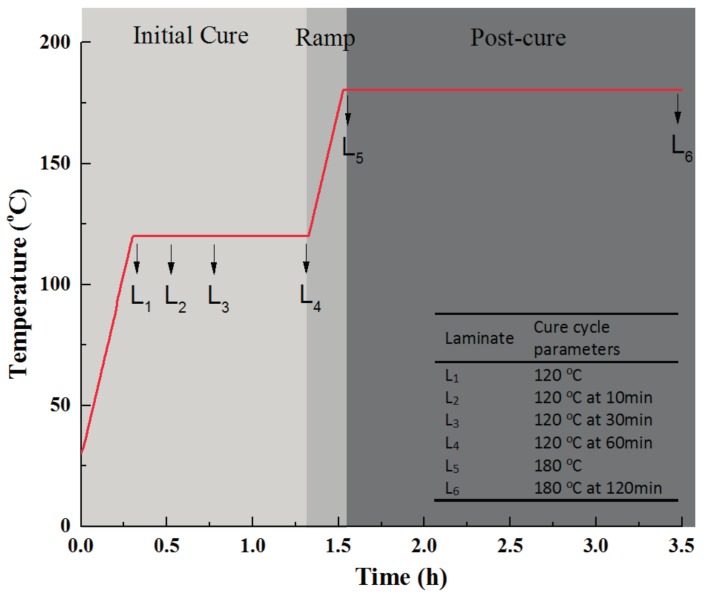
Laminates used to investigate resin impregnation evolution during rapidly cured process.

Six laminates were manufactured using the developed out-of-autoclave prepregs, which were labelled as L_1_, L_2_, L_3_, L_4_, L_5_, and L_6_. Since the resin impregnation mainly occurred at the initial cure stage, four laminates were made to evaluate the real condition at that stage. Another laminate was made when the temperature was raised to 180 °C to study the gelation of the resin. After the whole curing process, the other laminate was made to examine the final laminate quality. All laminates had the same stacking sequence of [0]_8_ and cured in the oven under the same vacuum condition. As shown in Figure 12, once the particular curing point was reached, the laminate was taken out and quickly cooled to freeze the real laminate microstructure at that time [31]. Samples were cut from various locations of the laminates, then mounted in epoxy resin, grounded, and polished. The micrographs of the samples were obtained by using a scanning electron microscope [32,33].

**Figure 13:**
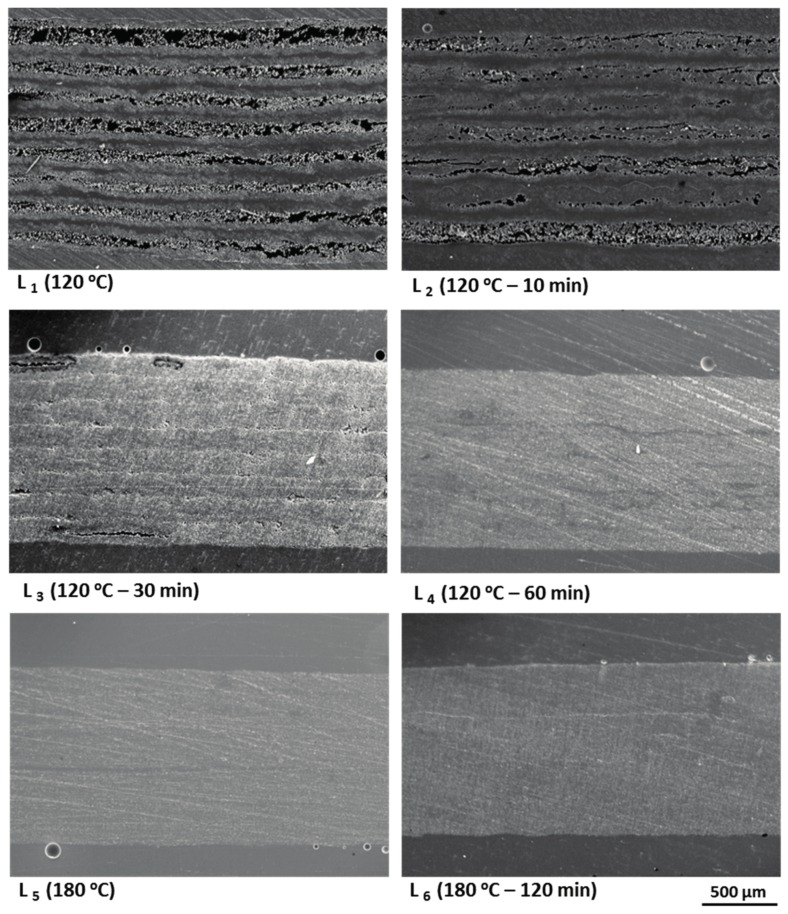
Optical micrographs of resin impregnation in laminates during a rapidly cured process.

Figure 13 shows the representative scanning results. It was revealed that at the beginning of 120 °C, apparent dry fiber areas as well as small circular voids between layers could be seen in the center of each layer. After dwelling at 120 °C for 10 min, the resin reached its minimum viscosity and infiltrated the dry fibers. Therefore, less dry areas existed in the laminate. When the resin infiltration lasted for 30 min at 120 °C, the driest fibers were impregnated and only partial linear dry areas and interlayer voids existed. After that, the resin further wet the partial dry fibers and the air in the interlayer voids gradually escaped from the thickness direction of the laminate. No obvious void was detected at the end of the initial cure stage, which the microstructure of L_4_ laminate showed. In addition, no void induced by volatiles was seen up to the post-curing stage, which was in agreement with the results from thermogravimetric analysis. Finally, the laminate achieved a good internal quality after the whole curing process, which was shown in L_6_.

**Figure 14:**
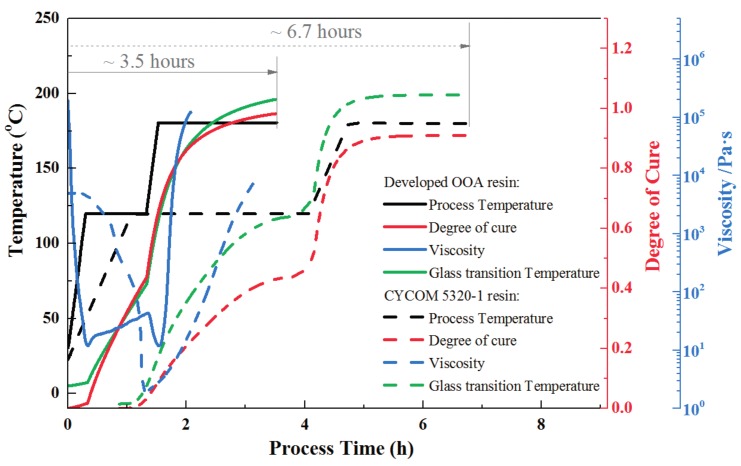
Manufacturing time saving of rapidly cured out-of-autoclave laminates compared with traditional out-of-autoclave products.

After tests, the degree of cure, viscosity, and glass transition temperature evolution of the developed prepreg during the rapid cure cycle were listed in Figure 14 and compared with those of traditional out-of-autoclave materials-CYCOM 5320-1 prepreg. The developed out-of-autoclave material showed an obvious high efficiency. The total manufacturing time for the developed resin was 3.5 h, which allowed for 48% saving of time compared to the 6.7 h of the traditional one. CYCOM5320-1 resin had a minimum viscosity of 2 Pa·s while the developed resin also had a relatively low resin viscosity for resin impregnation at the initial cure stage. After the cure cycle ended, both the developed and CYCOM5320-1 resin reached a high degree of cure, which was more than 0.9. In addition, the final glass transition temperature of CYCOM5320-1 composite was 212 °C while the developed composite had a competitive value of 205 °C where higher glass transition temperature represented better laminate quality.

**Figure 15:**
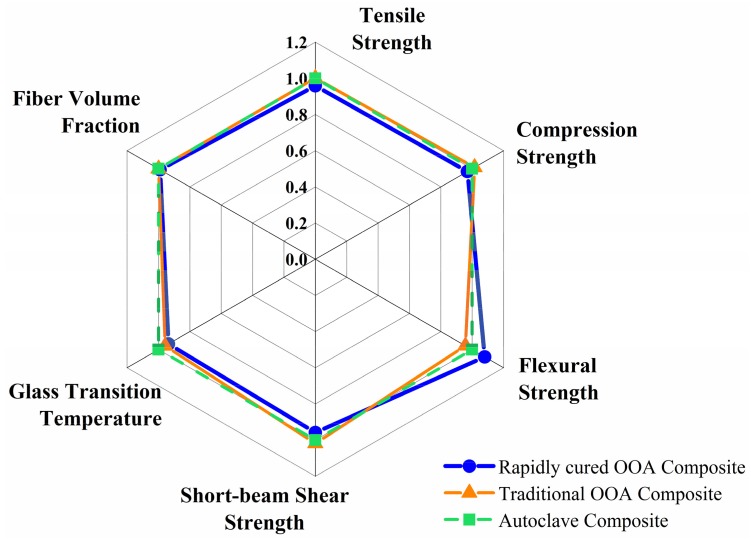
Quality examination of rapidly cured out-of-autoclave laminates compared with traditional out-of-autoclave and autoclave products.

Mechanical properties of the rapidly cured developed composites were evaluated and compared to those of traditional CYCOM5320-1 composites and autoclave composites, which is shown in Figure 15. The developed composite showed a fiber volume fraction of 59–60%, which is similar to that of traditional CYCOM5320-1 composites and autoclave composites.

The glass transition temperature of the developed and traditional out-of-autoclave composite was close while the autoclave composite had a somewhat higher temperature. The three types of composites had similar tensile strength due to the same fiber volume fraction since tensile strength was dominated by the fiber strength. In contrast, the compression strength was often affected by resin properties and fiber-resin bonding. The compression strength for the developed composite was 1658.1 MPa, which indicates the good cross-linking network and mechanical properties of the rapidly cured resin as well as the good bonding between fiber and resin. Moreover, the good interfacial bonding also contributed to the excellent flexural strength, which was 1906.2 MPa. It increased by 12.9% compared to that of a traditional out-of-autoclave composite and by 8.0% compared to that of autoclave composite. Additionally, the three types of composite had similar short-beam shear strength.

## 4. Conclusions

In this paper, a rapidly cured out-of-autoclave resin had been independently developed. Based on the resin, the typical partially impregnated out-of-autoclave prepregs reinforced with T800 carbon fibers were manufactured by a traditional two-step process. The effect of heating rate, initial cure temperature, dwelling time, and post-cure time on the final laminate quality was evaluated. A rapid optimized cure procedure was determined for the developed resin and the quality of laminate cured according to the determined cure cycle was examined.

Thermogravimetric analysis revealed that no weight degradation occurred under 200 °C for the developed prepreg, which indicates that the only factor for voids in the final laminate was insufficient air evacuation in this study. The degree of cure evolution results showed that the fast heating rate could save 35% cure time compared to the slow heating rate and the developed resin could be completely cured in the end. At the same time, the fast heating rate induced lower resin viscosity of 6.9 Pa·s, which was beneficial to resin infiltration in the out-of-autoclave process.

When the initial cure temperature was 120 °C, the resin flow time was more than 60 min, which was sufficient for resin infiltration before gelation at the initial cure stage. The resin gelation occurred fast at higher temperature and resin viscosity was not low enough for infiltration at lower temperature, which both lead to voids in the final laminates. As for the post-cure time, laminates post-cured for 2 h have a similar strength with that of laminates post-cured for 3 h. However, laminates post-cured for 1 h showed weak inter-laminar strength. The fracture modes and fracture surface also confirmed the results. 

Based on the results, an optimized rapid cure procedure was determined for the developed prepreg, which was dwelling at 120 °C for 1 h and was followed by dwelling at 180 °C for 2 h at a heating rate of 5 °C/min during the whole process. The results of the resin impregnation evolution during the cure cycle confirmed that, after resin infiltration for 30 min in the initial cure stage, the driest fibers were impregnated while only partial linear dry areas and interlayer voids existed. At the end of the initial cure stage, no obvious void was detected. Moreover, no void induced by volatiles was seen up to the post-cure stage. The laminate achieved good internal quality finally. In order to further validate the determined cure procedure, mechanical properties of the laminated were tested. The laminates using developed prepreg had a fiber volume fraction of 59–60% with a final glass transition temperature of 205 °C and excellent mechanical strength especially the flexural properties. 

The developed out-of-autoclave resin, relative prepreg, and composite can be rapidly cured, which saves 48% manufacturing time and still exhibits excellent mechanical properties compared to the traditional out-of-autoclave materials. However, further study needs to be done on the prepreg used for the complex and large scale structures. It is believed that the developed prepreg is a candidate of out-of-autoclave materials for industrial applications in the future.
